# Meetings of Societies

**Published:** 1932

**Authors:** 


### Meetings of Societies

### Bristol Medico-Chirurgical Society

The seventh meeting of the session was held in the Physiological Lecture Theatre of the University, on Wednesday, 13th April, at 8.30 p.m. Present: Dr. J. R. Charles in the Chair and seventy members.

The minutes of the previous meeting were read and confirmed.

Dr. F. J. Mayes showed skiagrams and Dr. A. L. Taylor the following pathological specimens: (*a*) Carcinoma of the œsophagus; (*b*) Diverticulitis; (*c*) Vertebræ from a case of multiple myeloma.

Dr. A. L. Taylor gave a short description and explanation of his specimens.

Mr. John Wright read a paper on "**The Management of the Ear, Nose and Throat in Influenza,**" reported on page 123.

The President congratulated Mr. Wright on his paper and declared the subject open for discussion.

Mr. E. Watson-Williams pointed out the variety of forms which influenza took, and instanced the recent epidemic, in which during the early months aural complications had been common, but more recently the nasal sinuses had been affected. He, in agreement with Mr. Wright, seldom operated in the acute phase. As a rule he avoided early paracentesis, but thought that in suppurative otitis media it minimized damage. He used magnesium sulphate in cases of otorrhœa and found it very valuable.

Mr. Angell James congratulated Mr. Wright and agreed with his views on treatment. However, he thought well of mercurochrome in spirit in the place of powder. He mentioned a recent case he had seen in which the tympanum had been full of blood. The case terminated fatally with a fulminating meningitis, and he wondered if early myringotomy would have been of value.

Dr. D. A. Alexander was glad to hear the specialists expressing their trust in Nature. He mentioned the loss of hair, smell and hearing which might occur in influenza.

Mr. G. R. SCARFF agreed with Mr. Wright. He did not like the use of watery antiseptics in the ear, and stressed the importance of the principle of osmosis. He found that hæmorrhagic bullæ often occurred in the external auditory meatus in fulminating cases.

Mr. OSMOND BODMAN never regretted early paracentesis. He combined glycerine and magnesium sulphate in the treatment of otorrhœa. He used inhalations of menthol, and preferred a douche can to a syringe in the treatment of tonsillitis. He suggested the use of anæsthetin as a gargle.

Dr. PARKER referred to the use of calomel and Mandl's paint.

The PRESIDENT stated that he had himself found great value and comfort from nasal douching during an acute sinusitis. He further suggested the use of chloroform as a gaseous antiseptic.

Mr. WRIGHT, in reply, affirmed his objection to douching the nose, and said he did not believe in gaseous antiseptics. He thought that the early deafness in influenza was a nerve defect. The prognosis in hæmorrhagic cases was always very bad.

Mr. E. R. CHAMBERS then read a paper on "**Some Common Ophthalmic Conditions,**" reported on page 131.

The PRESIDENT thanked Mr. Chambers for his paper and declared it open for discussion.

Mr. R. R. GARDEN mentioned the recent campaign against squint in Bristol, and stressed the importance of constant observations from an early age. He thought that any case in which there was frequent squint in one eye only twelve months needed treatment. The earliest at which he had prescribed glasses was fourteen months. He thought the occlusion disc useless, and agreed with pad and strapping.

Dr. H. ROGERS thought that true rheumatic iritis was still common, the cause being the streptococcus viridans.

Dr. C. A. H. GEE mentioned the common association of blepharitis with dandruff of the scalp.

Dr. A. T. F. ROWLEY said he found that parting the lids of a lusty infant was a very difficult work.

Mr. CHAMBERS, in reply, gave details of the method of holding a baby in order to treat its eyes.

The last meeting of the session was held in the Physiological Lecture Theatre of the University on Wednesday, 11th May, at 8.30 p.m.

Present, Dr. J. R. CHARLES in the Chair and fifty-two members.

The minutes of the previous meeting were read and confirmed.

Dr. G. B. BUSH showed skiagrams and Dr. FRASER pathological specimens.

Mr. A. W. ADAMS described a case of **Cerebello-pontine Tumour** and showed the brain from this case.

Dr. G. E. F. SUTTON read a paper on "**Hyperpiesia.**" A discussion followed in which the PRESIDENT, Mr. CHAMBERS, Dr. FRANK BODMAN, Dr. PERRY, Dr. WILSON-SMITH, Dr. PARKER spoke and Dr. SUTTON replied.

Mr. C. A. MOORE then read a paper entitled "**Some Notes on Surgical Jaundice,**" reported on page 139.

The PRESIDENT, Mr. DUNCAN WOOD and Dr. WILSON-SMITH discussed the paper, and Mr. MOORE replied.

